# Cellular therapy of the corneal stroma: a new type of corneal surgery for keratoconus and corneal dystrophies

**DOI:** 10.1186/s40662-018-0122-1

**Published:** 2018-11-01

**Authors:** Jorge L. Alió del Barrio, Jorge L. Alió

**Affiliations:** 1 Cornea, Cataract and Refractive Surgery Unit, Vissum Corporación, Alicante, Spain; 2 Division of Ophthalmology, Universidad Miguel Hernández, Alicante, Spain; 3 Vissum, Instituto Oftalmologico de Alicante, Avda de Denia s/n, 03016 Alicante, Spain

**Keywords:** Stem cells, Regenerative medicine, Corneal transplant, Decellularized cornea, Cornea, Corneal stroma, Cellular therapy, MSC, Mesenchymal stem cells

## Abstract

Cellular therapy of the corneal stroma, with either ocular or extraocular stem cells, has been gaining a lot of interest over the last decade. Multiple publications from different research groups are showing its potential benefits in relation to its capacity to improve or alleviate corneal scars, improve corneal transparency in metabolic diseases by enhancing the catabolism of the accumulated molecules, generate new organized collagen within the host stroma, and its immunosuppressive and immunomodulatory properties. Autologous extraocular stem cells do not require a healthy contralateral eye and they do not involve any ophthalmic procedures for their isolation. Mesenchymal stem cells have been the most widely assayed and have the best potential to differentiate into functional adult keratocytes in vivo and in vitro. While embryonic stem cells have been partially abandoned due to ethical implications, the discovery of the induced pluripotent stem cells (iPSC) has opened a new and very promising field for future research as they are pluripotent cells with the capacity to theoretically differentiate into any cell type, with the special advantage that they are obtained from adult differentiated cells. Cellular delivery into the corneal stroma has been experimentally assayed in vivo in multiple ways: systemic versus local injections with or without a carrier. Encouraging preliminary human clinical data is already available although still very limited, and further research is necessary in order to consolidate the clinical applications of this novel therapeutic line.

## Background

The stroma constitutes more than 90% of the corneal thickness. Many features of the cornea, including its strength, morphology and transparency are attributable to the anatomy and properties of the corneal stroma [1]. Many diseases such as corneal dystrophies, scars or ectatic disorders induce a distortion of its anatomy or physiology leading to loss of transparency and subsequent loss of vision. In the last decade, enormous efforts have been put into replicating the corneal stroma in the laboratory to find an alternative to classical corneal transplantation, but this has still not been accomplished due to the extreme difficulty in mimicking the highly complex ultrastructure of the corneal stroma, obtaining substitutes that either do not achieve enough transparency or strength [2, 3].

In the last few years, interest for cellular therapy of the corneal stroma using mesenchymal stem cells (MSCs) from either ocular or extraocular sources has gained a lot of interest; studies show that MSCs are capable of differentiating into adult keratocytes in vitro and in vivo [1]. Several authors, including reports from our research group, have demonstrated [4–6] that these stem cells can not only survive and differentiate into adult human keratocytes in xenogeneic scenarios without inducing any inflammatory reaction, but also i) produce new collagen within the host stroma [4, 7], ii) modulate preexisting scars by corneal stroma remodeling [8, 9], and iii) improve corneal transparency in animal models for corneal dystrophies by collagen reorganization as well as in animal models for metabolopathies by the catabolism of accumulated proteins [10–13]. Mesenchymal stem cells have also shown immunomodulatory properties in syngeneic, allogeneic and even xenogeneic scenarios [13, 14]. The first clinical data regarding the safety and preliminary efficacy of cellular therapy of the corneal stroma from Phase 1 human clinical trials is now available [15, 16], which may end up providing a real alternative treatment option for corneal diseases in the near future.

Considering existing scientific evidence, it seems that all types of MSCs have similar behavior in vivo [Table 1], and thus are able to achieve keratocyte differentiation and modulate the corneal stroma with immunomodulatory properties [17]. It has also been recently reported that MSCs secrete paracrine factors such as vascular endothelial growth factor (VEGF), platelet-derived growth factor (PDGF), hepatocyte growth factor (HGF), and transforming growth factor beta 1 (TGFβ1). Although the precise actions of the different growth factors for cornea wound healing are not fully understood, overall, they seem to promote cell migration, keratocyte survival by apoptosis inhibition, and upregulate the expression of extracellular matrix (ECM) component genes in keratocytes, subsequently enhancing corneal re-epithelialization and stromal wound healing [18]. MSCs can be obtained from many human tissues, including adipose tissue, bone marrow, umbilical cord, dental pulp, gingiva, hair follicle, cornea and placenta [19, 20].

**Table 1:** Stem cells assayed for corneal stroma regeneration: evidence of keratocyte or keratocyte-like differentiation and their potential autologous application

	CSSC	BM-MSC	ADASC	UMSC	ESC	iPSC
Keratocyte differentiation in vitro demonstrated	Yes	Yes	Yes	Yes	Yes	Yes
Keratocyte differentiation in vivo demonstrated	Yes	Yes	Yes	Yes	No	No
Possible autologous use	Yes/No	Yes	Yes	Yes/No	No	Yes

*CSSCs* = corneal stroma stem cells; *MSC* = mesenchymal stem cell; *BM* = bone marrow; *ADASC* = adipose-derived adult stem cell; *UMSC* = umbilical MSC; *ESC* = embryonic stem cell; *iPSC* = induced pluripotent stem cell

Corneal stroma stem cells (CSSCs) are a promising source for cellular therapy as the isolation technique and culture methods have been optimized and refined [21]; presumably, they should be efficient in differentiating into keratocytes as they are already committed to the corneal lineage. On the other hand, isolating CSSCs autologously is more technically demanding considering the small amount of tissue that they are obtained from. Furthermore, this technique still requires a contralateral healthy eye, which is not always available (bilateral disease). Therefore, these drawbacks may limit its use in clinical practice. Allogeneic CSSC use requires living or cadaveric donor corneal tissue.

Human adult adipose tissue is a good source of autologous extraocular stem cells as it satisfies many requirements: easy accessibility to the tissue, high cell retrieval efficiency and the ability of its stem cells (h-ADASCs) to differentiate into multiple cell types (keratocytes, osteoblasts, chondroblasts, myoblasts, hepatocytes, neurons, etc.) [4]. This cellular differentiation occurs due to the effect of very specific stimulating factors or environments for each cell type, avoiding the mix of multiple kinds of cells in different niches.

Bone marrow MSCs (BM-MSCs) are the most widely studied MSCs, presenting a similar profile to ADASCs, but their extraction requires a bone marrow puncture, which is a complicated and painful procedure requiring general anesthesia.

Umbilical MSCs (UMSCs) present an attractive alternative, but their autologous use is currently limited as the umbilical cord is not generally stored after birth.

Embryonic stem cells have great potential, but also present important ethical issues. However, the use of iPSC technology [22] could solve such problems, and their capability to generate adult keratocytes has already been proven in vitro [23].

Finally, it is important to remark that the therapeutic effect of MSCs in a damaged tissue is not always related to the potential differentiation of the MSCs in the host tissue as multiple mechanisms might contribute simultaneously to this therapeutic action for example, secretion of paracrine trophic and growth factors capable of stimulating resident stem cells, reduction of tissue injury and activation of immunomodulatory effects, in which case the direct cellular differentiation of the MSCs might not be relevant and could even be non-existent [17, 24, 25].

We will review the different types of stem cells (mesenchymal and others) that have been proposed for the regeneration of the corneal stroma as well as the current in vitro or in vivo evidence. Finally, we will review the different surgical approaches that have been suggested (in vivo) for the application of stem cell therapy to regenerate the corneal stroma.

## Main text

### Stem cell sources used for corneal stroma regeneration

#### Bone marrow mesenchymal stem cells (BM-MSCs)

Park et al. reported that human BM-MSCs differentiate in vitro into keratocyte-like cells when they are grown in specific keratocyte differentiation conditions [26]. They demonstrated a strong expression of keratocyte markers such as lumican and ALDH (aldehyde dehydrogenase) along with the loss of expression of MSC markers such as α-smooth muscle actin. However, they could not demonstrate an evident expression of keratocan on these differentiated cells [26]. Trosan et al. showed that mice BM-MSCs cultured in corneal extracts and insulin-like growth factor-I (IGF-I), efficiently differentiate into corneal-like cells with expression of corneal specific markers, such as cytokeratin 12, keratocan, and lumican [27]. The survival and differentiation of human BM-MSCs into keratocytes has also been demonstrated in vivo when these cells are transplanted inside the corneal stroma. Keratocan expression was observed without any sign of immune or inflammatory response [28].

#### Adipose-derived adult mesenchymal stem cells (ADASCs)

**Fig. 1:**
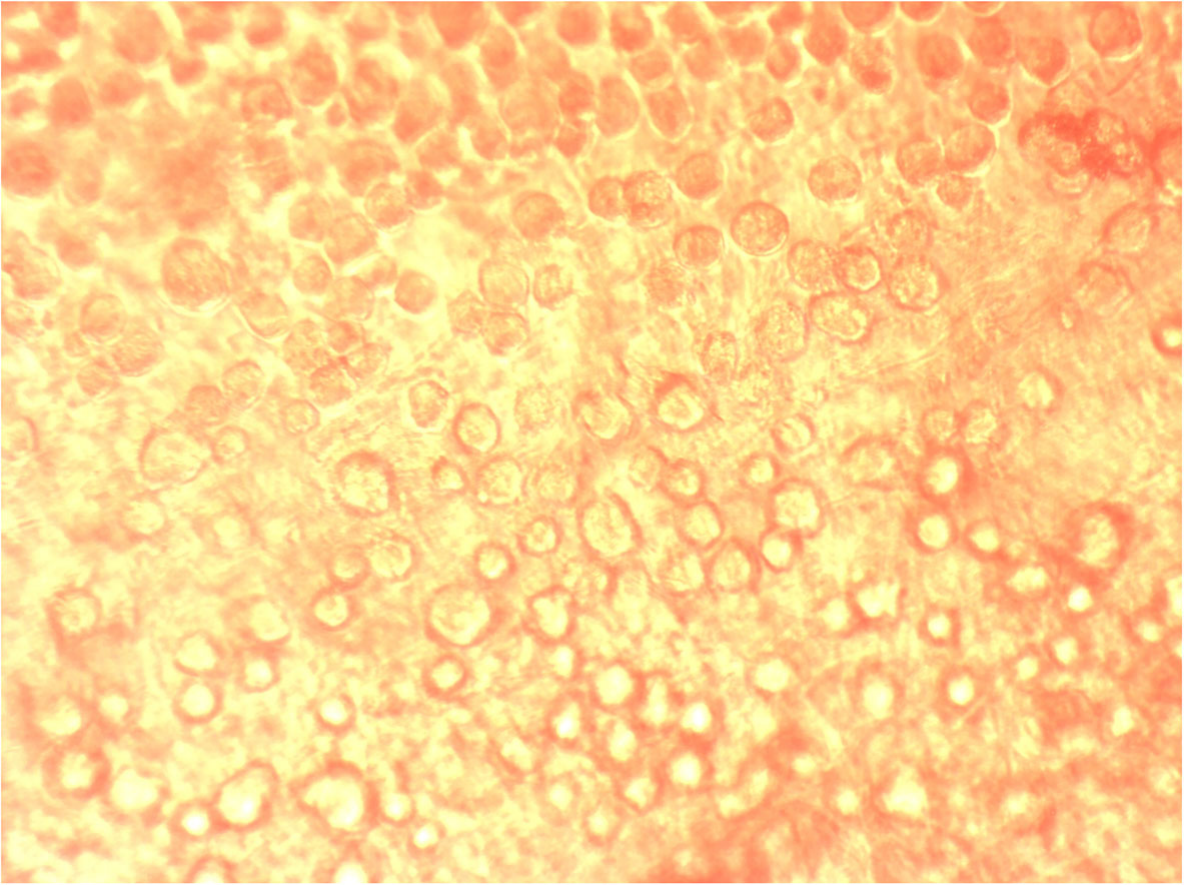
Microscopic appearance (phase-contrast photograph) of human ADASCs (10× magnification)

Human ADASCs (h-ADASCs) cultured in vitro (Fig. 1) under keratocyte differentiation conditions express collagens and other corneal-specific matrix components. This expression is quantitatively similar to that achieved by differentiated h-CSSCs [29].

The differentiation of h-ADASCs in functional human keratocytes has also been demonstrated in vivo, for the first time, in a previous study by our group using the rabbit as a model [4]. These cells, once implanted intrastromally, express not only collagens type I and VI (the main components of corneal extracellular matrix), but also keratocyte specific markers such as keratocan or ALDH, without inducing an immune or inflammatory response. These findings were later reproduced and confirmed by other authors in several research papers [17].

#### Umbilical cord mesenchymal stem cells (UCMSCs)

Human MSCs isolated from neonatal umbilical cords have exhibited similar differentiation behavior to other types of MSCs when transplanted inside the corneal stroma in vivo, expressing keratocyte-specific markers such as keratocan without inducing immune or rejection responses [30]. Liu et al. reported that the injection of these cells inside the corneal stroma of lumican null mice improved corneal transparency and increased stromal thickness with a reorganized collagen lamellae, and also improved host keratocyte function through enhanced expression of keratocan and ALDH in these mice [11]. These data are encouraging, although to date, autologous use of UCMSCs is not possible as the umbilical cord from new births is not generally stored.

#### Embryonic stem cells (ESCs)

Current experience with these human pluripotent stem cells for the corneal stroma regeneration is much more limited. Chan et al. reported that differentiation of these cells into a keratocyte lineage can be induced in vitro, demonstrating an upregulation of keratocyte markers including keratocan [31].

To the best of our knowledge, no in vivo studies with these cells have been performed in the field of regenerative medicine for the corneal stroma. The use of these cells also raises many ethical issues, and together with the lack of in vivo data, discourages their current use in a clinical setting.

#### Induced pluripotent stem cells (iPSCs)

As already discussed, the use of embryonic stem cells has been partially abandoned because of ethical concerns and especially since the discovery of iPSCs [22], which are derived from adult cells. In 2012, Shinya Yamanaka from Japan and John B. Gurdon from the UK received the Nobel Prize for Medicine for discovering that mature, specialized cells can be reprogrammed to an immature or stem cell state and then redirected to the required cell lineage using specific factors and environmental stimuli. iPSCs promise to be the future of tissue and cellular engineering.

Regarding their application in the regeneration of the corneal stroma, human iPSCs have shown the capability to differentiate into neural crest cells (the embryonic precursor to keratocytes). By culturing them on cadaveric corneal tissue, it promotes their keratocyte differentiation by acquiring a keratocyte-like morphology to express markers similar to corneal keratocytes [23]. It has also been shown that iPSC-derived MSCs exert immunomodulatory properties in the cornea similar to those observed with BM-MSCs [32]. To the best of our knowledge, no studies have been published reporting the capability of iPSCs to differentiate into adult keratocytes in vivo in the animal model.

#### Corneal stromal stem cells (CSSCs)

The limbal palisades of Vogt form a niche that contains both limbal epithelial stem cells (LESCs) and corneal stromal stem cells (CSSCs) [33]. CSSCs express genes typical of descendants of the neural ectoderm such as PAX6, adult stem cell markers such as ABCG2 and MSC markers such as CD73 and CD90 [33, 34]. They exhibit clonal growth, self-renewal properties and a potential for differentiation into multiple distinct cell types. Unlike keratocytes, human CSSCs (h-CSSCs) undergo extensive expansion in vitro without losing their ability to adopt a keratocyte phenotype [33, 34]. These corneal MSCs have a demonstrated potential for differentiation into corneal epithelium and adult keratocytes in vitro [33, 35]. When cultured on a substratum of parallel aligned polymeric nanofibers, h-CSSCs produce layers of highly parallel collagen fibers with packing and fibril diameter indistinguishable from that of the human stromal lamellae [36]. The ability of h-CSSCs to adopt a keratocyte function has been even more striking in vivo. When injected into the mouse corneal stroma, h-CSSCs express keratocyte mRNA and protein, replacing the mouse ECM with human matrix components. These injected cells remain viable for many months, apparently becoming quiescent keratocytes [10].

All these experimental data have raised interest in this novel cell-based therapy for corneal stromal diseases, however, before its application in clinical practice, its efficacy and safety need to be well proven in human clinical trials, while other limitations such as the high laboratory costs and potential therapeutic efficacy differences among different donors have to be given serious consideration.

### Corneal stroma regeneration techniques

All these types of stem cells have been used in various ways in several research projects in order to find the optimal procedure to regenerate the human corneal stroma. Corneal MSC implantation has been assayed and studied by direct intrastromal transplantation or after implantation from the ocular surface, intravenously and the anterior chamber where cellular migration within the stroma is to be expected. Different cellular carriers have been analyzed in order to enhance the potential benefits of this therapy.

#### Ocular surface implantation of stem cells

Surface implantation of MSCs would be the optimal approach for ocular surface reconstruction and corneal epithelium/limbal stem cell niche regeneration (not the aim of this review). However, surface implantation of MSCs would still play a role in the prevention or modulation of anterior stromal scars after an ocular surface injury (like a chemical burn). As discussed previously, MSCs secrete paracrine factors that enhance corneal re-epithelialization and stromal wound healing [18]. Thus, the benefit of MSCs on the ocular surface may be more justified by these paracrine effects rather than by direct differentiation of the MSCs into epithelial cells, as the evidence for the latter is controversial. In this respect, Di et al. assayed *subconjunctival injections* of BM-MSCs in diabetic mice and reported an increased corneal epithelial cell proliferation as well as an attenuated inflammatory response mediated by tumor necrosis factor-α-stimulated gene 6 (TSG6) [37].

Holan et al. suggested MSC application on the ocular surface using *nanofiber scaffolds*. They reported that BM-MSCs grown on these scaffolds can enhance re-epithelialization, suppress neovascularization and local inflammatory reaction when applied on an alkali-injured eye in a rabbit model, and these results were comparable to those obtained with limbal epithelial stem cells, and both were better than those obtained with ADASCs [38]. The same group suggested that these results can be improved when these nanofiber scaffolds seeded with rabbit BM-MSCs are covered with cyclosporine-A (CSA) loaded nanofiber scaffolds, observing an even greater scar suppression and healing results with the combination of both nanofibers (MSC and CSA) [39].

*Topical application* of a suspension of autologous ADASCs has been reported in an isolated clinical case report where authors describe the healing of a neurotrophic ulcer that is not responsive to conventional treatment [40]. The lack of further scientific evidence for this delivery method since 2012 raises questions about its real efficacy.

Finally, Basu et al. suggested the delivery of MSCs using *fibrin glue* [21]. They resuspended CSSCs in a solution of human fibrinogen, and this was added onto a wounded ocular surface with thrombin on the wound bed. Subsequently the fibrinogen gels. Using this application, they demonstrated the prevention of corneal scarring in the mouse model together with the generation of new stroma with a collagen organization indistinguishable from that of native tissue. Currently, this group is enrolled in a clinical trial to validate these findings, using autologous and heterologous CSSCs from limbal biopsies for cases of chemical burns, neurotrophic ulcers and established scars. Preliminary report showed an improvement in visual parameters, corneal epithelization, corneal neovascularization and corneal clarity [41].

#### Intrastromal implantation of stem cells alone

Direct in vivo injection of stem cells inside the corneal stroma has been assayed in some studies, demonstrating the differentiation of stem cells into adult keratocytes without signs of immune rejection. In our study, we also demonstrated the production of human ECM by immunohistochemistry when h-ADASCs were transplanted inside the rabbit cornea [4]. As expected, collagen types I and VI were found to be expressed in the rabbits’ corneal stroma as well as in the transplanted h-ADASCs; collagen types III and IV, not normally expressed in the corneal stroma, were not detected either in the host corneal stroma or in the transplanted h-ADASCs. Du et al. [10] reported restoration of corneal transparency and thickness in lumican null mice (thin corneas, haze and disruption of normal stromal organization) 3 months after intrastromal transplant of human CSSCs. They also confirmed that human keratan sulphate was deposited in the mouse stroma and the host collagen lamellae were reorganized, concluding that delivery of h-CSSCs to the scarred human stroma may alleviate corneal scars without requiring surgery [10]. Very similar findings were reported by Liu et al. who utilized h-UMSCs using the same animal model [11]. Coulson-Thomas et al. found that, in a mouse model for mucopolysaccharidosis, transplanted h-UMSCs participate both in extracellular glycosaminoglycans (GAG) turnover and enable host keratocytes to catabolize accumulated GAG products [12].

**Fig. 2:**
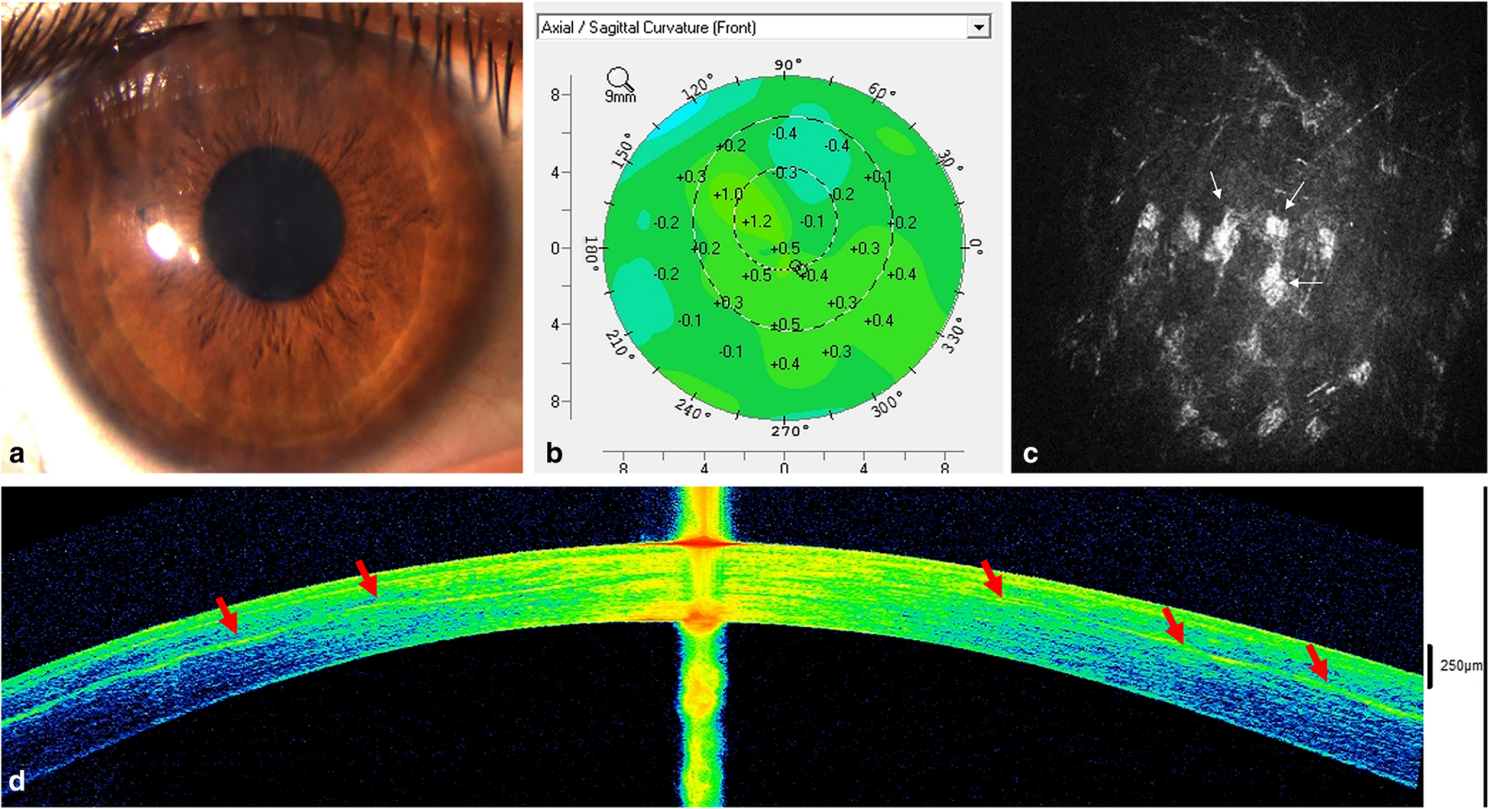
Autologous h-ADASC corneal stroma implantation for advanced keratoconus. **a**) Slit lamp picture one year after the procedure; **b**) Topographic changes (Pentacam) between preop and 12 months after surgery. Observe the stability of the keratometric parameters; **c**) Corneal confocal biomicroscopy pictures at the surgical plane in the first postoperative month. Stem cell survival is confirmed by the presence of cells showing a more rounded morphology (white arrows) (image corresponds to an area of 100 × 100 μm); **d**) AS-OCT picture 1 year postoperatively. Note the patched hyper-reflective areas (red arrows) at the level of the stromal pocket compatible with areas of new collagen production

Recently, our group has published the first clinical trial in which the preliminary safety and efficacy of the cellular therapy of the human corneal stroma is reported [15, 42]. In this pilot clinical trial, we implanted autologous ADASCs (obtained by elective liposuction) in a mid-stroma femtosecond laser-assisted lamellar pocket in patients with advanced keratoconus (already candidates for corneal transplantation; *n* = 5). No signs of inflammation or rejection were observed, confirming all previous evidence reported in the animal model. We also demonstrated, by anterior segment optical coherence tomography (AS-OCT), the production of new collagen in the area of the MSC implantation (Fig. 2), with a mean central pachymetric improvement of 15 μm. This neo-collagen band did not induce any clinical haze (Fig. 2), and all patients moderately improved their visual function (mean improvement of 2 lines in all visual parameters), while keratometric values remained stable (Fig. 2) [15, 42]. Further studies with larger samples are required in order to confirm this preliminary data, but encouraging results were observed, thus opening a new and exciting line of therapy for research. As mentioned, the production of new ECM by the implanted MSCs occurs, but is not quantitatively enough to be able to restore the thickness of a severely diseased human cornea (as in extreme keratoconic corneas). However, the direct injection of stem cells may provide a promising treatment modality for corneal dystrophies, corneal stroma progressive opacification in the context of systemic metabolic disorders, and for the modulation of corneal scarring.

#### Intrastromal implantation of stem cells together with a biodegradable scaffold

To enhance the growth and development of the stem cells injected into the corneal stroma, transplantation together with biodegradable synthetic ECM has been performed. Espandar et al. injected h-ADASCs with a semisolid hyaluronic acid hydrogel into the rabbit corneal stroma and reported better survival and keratocyte differentiation of the h-ADASCs when compared with their injection alone [7]. Ma et al. used rabbit ADSCs with a polylactic-co-glycolic (PLGA) biodegradable scaffold in a rabbit model of stromal injury wherein they observed newly formed tissue with successful collagen remodeling and less stromal scarring [43]. Initial data show that these scaffolds could enhance stem cell effects on corneal stroma, although further research is required and warranted.

#### Intrastromal implantation of stem cells with a Decellularized corneal stroma scaffold

The complex structure of the corneal stroma has still not been replicated and there are well-known drawbacks to the use of synthetic scaffold-based designs: i) strong inflammatory responses induced on their biodegradation and ii) nearly all polymer materials cause a nonspecific inflammatory response [5].

**Fig. 3:**
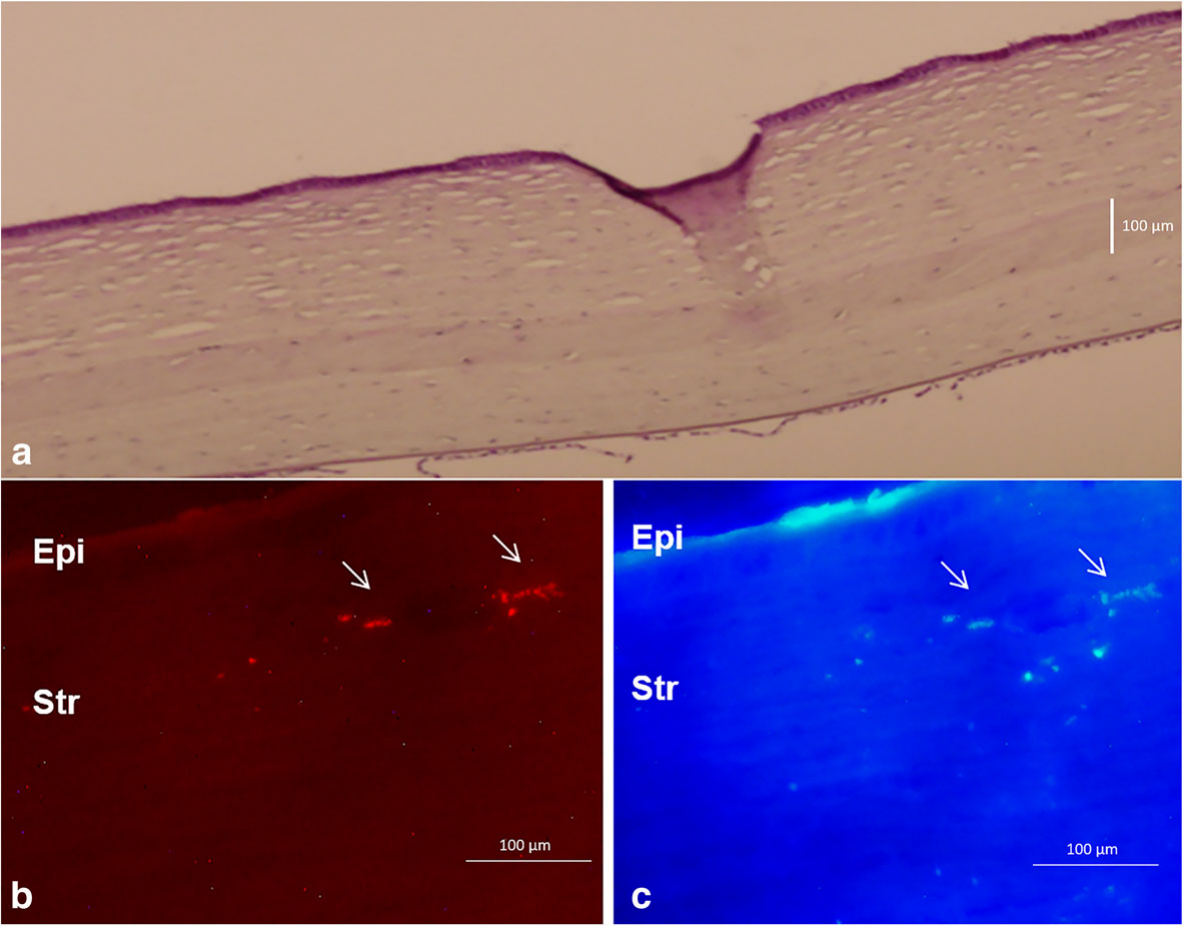
Corneal stroma enhancement with decellularized human corneal stroma with h-ADASC recellularization in the rabbit animal model. **a** Hematoxylin-eosin staining of a rabbit cornea with an implanted graft of decellularized human corneal stroma with h-ADASC colonization. (magnification 200×); **b**) Human cells (arrows), labelled with CM-DiI, around and inside the implant confirming the presence of living human cells inside the rabbit corneal stroma; **c**) Same section showing human keratocan and their eventual differentiation into human keratocytes (arrows) (magnification 400×); Abbreviations: Epi: epithelium; Str: stroma

Recently, several corneal decellularization techniques have been described, which provide an acellular corneal ECM [44]. These scaffolds have gained attention in the last few years as they provide a more natural environment for the growth and differentiation of cells when compared with synthetic scaffolds. In addition, components of the ECM are generally conserved among species and are well tolerated by xenogeneic recipients. Moreover, keratocytes are essential for remodeling the corneal stroma and for normal epithelial physiology [45]. This highlights the importance of transplanting a cellular substitute together with the structural support (acellular ECM) to undertake these critical functions in corneal homeostasis. To the best of our knowledge, all attempts to repopulate decellularized corneal scaffolds have used corneal cells [46–48], but as already discussed, these cells have significant drawbacks that limit their autologous use in clinical practice (damage to the donor tissue, lack of cells and more difficult cell subcultures), thus redirecting efforts to find an extraocular source of autologous cells. In a previous study by our group, we showed the perfect biointegration of human decellularized corneal stromal sheets (100 μm thickness) with and without h-ADASC colonization inside the rabbit cornea (Fig. 3a) and observed no rejection response despite the graft being xenogeneic [6]. We also demonstrated the differentiation of h-ADASCs into functional keratocytes inside these implants in vivo, which then achieved their proper biofunctionalization (Fig. 3b-c). In our experience, decellularization of the whole (~ 500 μm) corneal stroma (using sodium dodecyl sulfate anionic detergent) lacks efficacy, as it not possible to completely remove the whole cellular component. However, we demonstrated that this method completely removes the cellular component and preserves the tissue integrity of the corneal stroma when thinner lenticules are treated; a method that has been later confirmed by other authors with the use of electron microscopy [6, 49, 50]. Others have also assayed the integration of decellularized pig articular cartilage ECM colonized with mice BM-MSCs in the rabbit corneal stroma and reported similar findings, although the transparency of these decellularized scaffolds was not clearly reported [51].

**Fig. 4:**
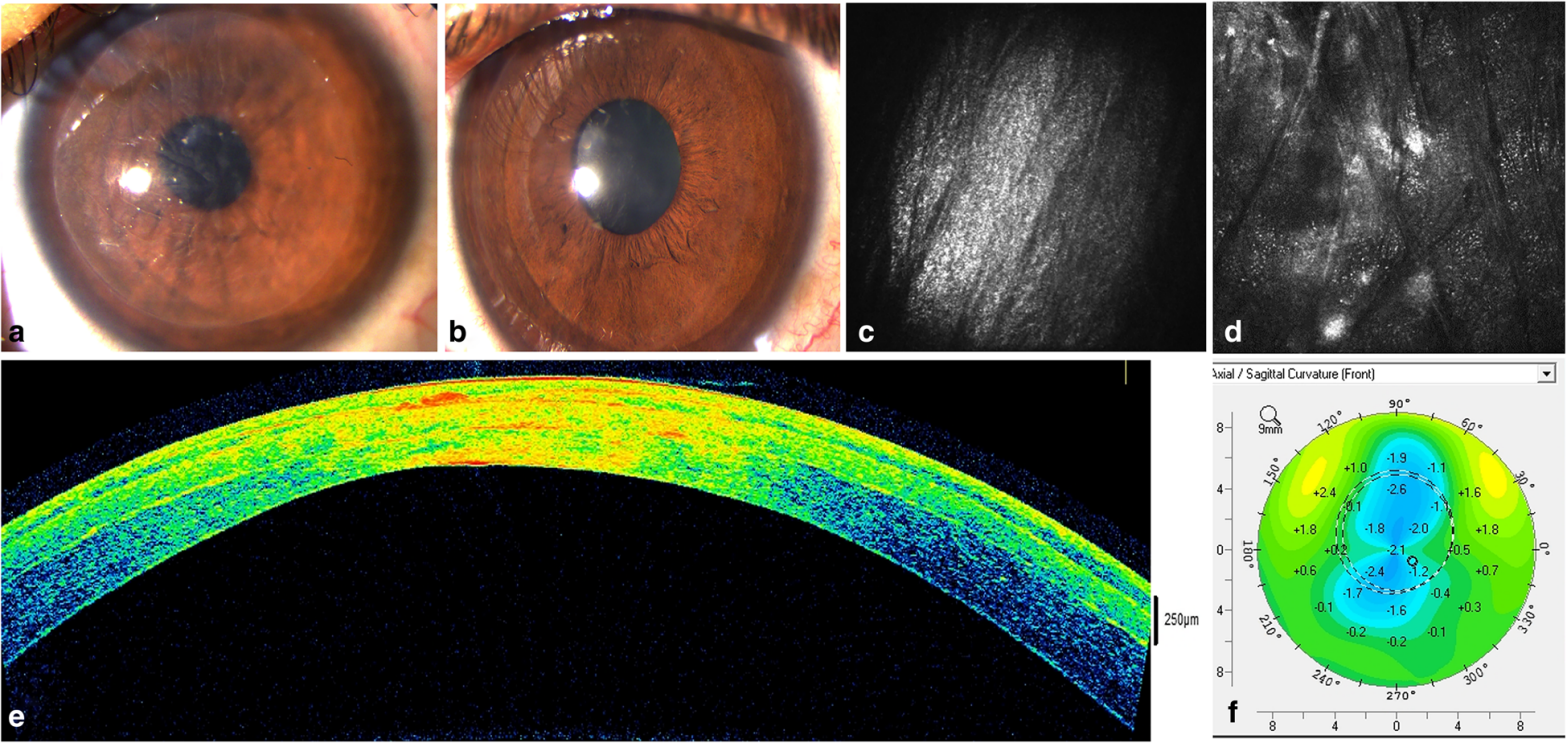
Corneal stroma enhancement with decellularized human corneal stroma with or without autologous h-ADASC recellularization. **a**-**b** Slit lamp pictures 1 week (A) and 3 months (B) after surgery. Observe the complete restoration of corneal transparency; **c**) Corneal confocal biomicroscopy picture. The implanted lamina showed a complete acellular pattern 3 months after surgery; **d**) Recellularization signs of the implanted lamina 12 months after surgery (images correspond to an area of 100 × 100 μm); **e**) AS-OCT image one year after corneal stroma enhancement; **f**) Topographic changes between preop and 12 months after surgery. Observe the significant flattening of the keratometry

In our opinion, the implantation of MSCs together with decellularized corneal ECM would be the best technique to effectively restore the thickness of a diseased and severely weakened human cornea because the implantation of MSCs alone only achieves a limited new ECM formation and thickness restoration [4, 15]. Moreover, through this technique, and by using autologous MSCs from a given patient, it is theoretically possible to transform allogenic grafts into functional autologous grafts, thus avoiding any risk of rejection. Following this research line, we have recently published the first clinical trial using these decellularized human corneal stroma scaffolds (120 μm thickness and 9.0 mm diameter laminas), with or without autologous ADASC recellularization, in patients with advanced keratoconus [16, 42]. We demonstrated a moderate but significant improvement in all visual parameters (about two lines of improvement), together with a reduction in refractive sphere, significant anterior keratometric flattening (Fig. 4f), and improvement in corneal aberrometry, especially the spherical aberration [16, 42]. Complete corneal transparency without significant haze was obtained 3 months after the procedure (Fig. 4a-b), and all pachymetric parameters improved over 100 μm as expected, anatomically restoring these advanced keratoconic corneas (Fig. 4e). Although no clinical differences could be demonstrated between decellularized and recellularized implants, the host keratocyte recellularization of the implants and the increase in the keratocyte density in the anterior and posterior stroma was more intense in those patients where autologous ADASCs were also implanted (Fig. 4c-d) [16, 42].

Decellularized tissues have the drawback of requiring specific laboratory equipment, although eye banks could potentially do it and deliver such grafts to different clinical centers. Keratophakia (intrastromal insertion of an allogeneic lenticule) was described by Barraquer in 1964, but was abandoned due to the unpredictability of the refractive outcome and the relatively high frequency of interface haze development [52]. The lack of haze observed in our pilot clinical trial could be in relation with the absence of donor keratocytes that could potentially activate postoperatively and generate scar tissue. Moreover, rejection episodes have already been described after the implantation of allogeneic lenticules, a risk that is theoretically avoided by the use of decellularized grafts [50]. We should consider that as long as human decellularized tissue is used, there will be no risk for zoonotic diseases.

#### Anterior chamber injection of stem cells

Demirayak et al. reported that BM-MSCs and ADASCs, suspended in phosphate-buffered solution (PBS) and injected into the anterior chamber after a penetrating corneal injury in a mouse model, are able to colonize the corneal stroma and increase the expression of keratocyte specific markers such as keratocan, with a demonstrated increase in keratocyte density by confocal microscopy [9]. Conversely, the possible side effects of this MSC injection into the anterior chamber for the lens epithelium and trabecullum is highly questionable as it may induce scarring and a subsequent glaucoma. Considering this, the potential clinical use of this approach, in our opinion, is limited.

#### Intravenous injection of stem cells

Systemic use, by intravenous injection, of MSCs has also been tested. Intravenous injection of BM-MSCs in mice after an allograft corneal transplant was be able to colonize the transplanted cornea and conjunctiva (inflamed ocular tissues) but not the contralateral ungrafted cornea, simultaneously decreasing immunity and significantly improving allograft survival rate [53]. Yun et al. recently reported similar findings with the intravenous injection of iPSC-derived MSCs and BM-MSCs after a surface chemical injury, where they observed that the corneal opacity, inflammatory infiltration and inflammatory markers in the cornea were markedly decreased in the treated mice, without significant differences between both MSC types [32]. In contrast, our group did not observe any benefit in corneal allograft survival and rejection rates after systemic injection of rabbit ADASCs prior to surgery, during surgery, and at various times after surgery in rabbits with vascularized corneas (model more similar to human corneal transplants than those reported in mice). A shorter graft survival compared with the non-treated corneal grafts was noted [54].

### Autologous versus allogenic MSC

A critical question for future clinical trials to further assess the feasibility of cellular therapy of the corneal stroma, is whether the use of autologous MSCs is really necessary and whether allogenic MSCs could achieve the same benefit without any risk of inflammation or rejection. If we consider all published evidence in the animal model where human MSCs were implanted in the corneal stroma, despite being a xenogeneic transplant, no signs of rejection or inflammation have ever been reported [4–13]. This coincides with the strong evidence regarding the immunomodulatory and immunosuppressive properties of MSCs, which help them to evade host immune rejection and survive by inhibiting adhesion and invasion, and inducing cell death of inflammatory cells, partially due to a rich extracellular glycocalyx that contains tumor necrosis factor-α-stimulated gene 6 (TSG6) [14, 55]. TSG6 has been demonstrated to play a critical role in the immunosuppressive properties exhibited by MSCs [13, 32, 37]. Taken together, the use of allogenic MSCs would greatly simplify the clinical application of MSCs, as clinical application centers would not need any specific equipment because potential MSC banks could store and supply stem cells for their use in patients. There are already low-cost systems available that are capable of enhancing the preservation of MSCs at hypothermic temperatures, while maintaining their normal function, thereby widening the time frame for distribution between the manufacturing site and the clinic, and reducing the waste associated with the limited shelf life of cells stored in their liquid state [56]. Funderburgh et al. recently reported that MSCs from different donors may have different immunosuppressive properties, and consequently, different abilities to regenerate and relieve stromal scars [57]. Considering this important finding, the best donors could be selected by MSC banks in order to expand and supply only those MSCs with the highest immunosuppressive and regenerative capacity, so autologous cells would not be necessary. We should also consider that adult keratocytes obtained from autologous MSCs may still carry the same genetic defect that led to the corneal disease such as in the case of corneal dystrophy. In this scenario, the use of allogenic instead of autologous MSCs would be interesting. A recent study observed gene expression differences between the iPSC-derived keratocytes generated from fibroblasts of both keratoconic and normal human corneal stroma, influencing cellular growth and proliferation, confirming that, at least in keratoconus cases, adult cells obtained from MSCs may still not be functionally normal [58].

### MSC

#### Exosomes

Exosomes are nano-sized extracellular vesicles that originate from the fusion of intracellular multivesicular bodies with cell membranes and are released into extracellular spaces [55]. They have been implicated in the ability of MSCs to repair damaged tissue. Funderburgh et al. recently showed that exosomes isolated from the culture media of human CSSCs had similar immunosuppressive properties and also significantly reduced stromal scarring in wounded corneas in vivo [59]. This finding suggests that for some diseases, such as prevention or reduction of corneal scars, MSC exosomes may provide a non-cell based therapy [57]. Zhang et al. suggested that exosomes released by transplanted UCMSCs in the diseased cornea are able to enter into host corneal keratocytes and endothelial cells and enhance their functions [55]. In their in vitro experiment using mucopolysaccharidosis VII mice, they discovered that UCMSC-secreted exosomes assisted in the recycling process of accumulated glycosaminoglycans (GAGS) in the lysosomes of diseased cells [12]. These findings open an exciting new field for research as the use of exosomes per se could overcome some of the limitations and risks associated to intrastromal cellular injection, given that exosomes can be potentially applied topically [30].

## Conclusions

In conclusion, cellular therapy of the corneal stroma is a novel treatment modality for stromal diseases, which even though further studies are still required in the form of clinical trials with larger sample sizes in order to definitely establish its safety and efficacy for different stromal diseases, the initial results obtained from the first few pilot clinical trials are encouraging. In our opinion, the creation of cellular banks storing and expanding stem cells or their exosomes, and their shipping and delivery to the different clinical centers for their use may be the future for this promising treatment modality, although prior to this, there is still a lot of research work to be undertaken.
